# Feasibility study of a clinical decision support system for the management of multimorbid seniors in primary care: study protocol

**DOI:** 10.1186/s40814-016-0057-z

**Published:** 2016-03-12

**Authors:** Birgitta Weltermann, Christine Kersting

**Affiliations:** Institute for General Medicine, University Hospital Essen, University of Duisburg-Essen, Hufelandstraße 55, 45147 Essen, Germany

**Keywords:** Multimorbidity, Primary health care, Comprehensive health care, Clinical decision support system, Feasibility study

## Abstract

**Background:**

Care for seniors is complex because patients often have more than one disease, one medication, and one physician. It is a key challenge for primary care physicians to structure the various aspects of each patient’s care, to integrate each patient’s preferences, and to maintain a long-term overview. This article describes the design for the development and feasibility testing of the clinical decision support system (CDSS) eCare*Seniors© which is electronic health record (EHR)-based allowing for a long-term, comprehensive, evidence-based, and patient preference-oriented management of multimorbid seniors.

**Methods/design:**

This mixed-methods study is designed in three steps. First, focus groups and practice observations will be conducted to develop criteria for software design from a physicians’ and practice assistants’ perspective. Second, based on these criteria, a CDSS prototype will be developed. Third, the prototype’s feasibility will be tested by five primary care practices in the care of 30 multimorbid seniors. Primary outcome is the usability of the software measured by the validated system usability scale (SUS) after 3 months. Secondary outcomes are the (a) willingness to routinely use the CDSS, (b) degree of utilization of the CDSS, (c) acceptance of the CDSS, (d) willingness of the physicians to purchase the CDSS, and (e) willingness of the practice assistants to use the CDSS in the long term. These outcomes will be measured using semi-structured interviews and software usage data. If the SUS score reaches ≥70 %, feasibility testing will be judged successful. Otherwise, the CDSS prototype will be refined according to the users’ needs and retested by the physicians and practice assistants until it is fully adapted to their requirements and reaches a usability score ≥70 %.

**Discussion:**

The study will support the development of a CDSS which is primary care-defined, user-friendly, easy-to-comprehend, workflow-oriented, and comprehensive. The software will assist physicians and practices in their long-term, individualized care for multimorbid seniors.

**Trial registration:**

German Clinical Trials Register, DRKS00008777

## Background

Primary care for seniors is complex because patients often have more than one disease, one medication, and one physician. Population-based studies show that 55–98 % of seniors (≥65 years of age) exhibit multimorbidity [1], meaning that they have more than one chronic disease [2]. This disease burden results in an average intake of 3.6 different pharmacological agents per day: 33 % of the seniors meet the criteria for polypharmacy (defined by the World Health Organization as five or more substances) and 65 % of them take five to seven different agents daily [3]. The medications most frequently prescribed in this age group are used for widespread diseases such as hypertension, coronary heart disease, cardiac insufficiency, chronic stomach problems, and diabetes [4]. From a physician’s perspective, the demographic development with the aging of populations, the differentiation of modern medicine with evolving therapeutic options, and the diversity of patients’ choices increase the complexity of health care management for this age group.

A survey among primary care physicians showed that physicians are willing to accept a clinical decision support system (CDSS) when it is designed to support their care for elderly patients with multiple chronic diseases and/or polypharmacy [5]. Yet, although medical informatics is considered to have this potential, the current practice administration systems are typically not geared to handle this complexity as they focus on billing and treatment documentation, while their orientation on complexity, quality of care, and longitudinal personalized care is poor. Existing CDSSs showed inconsistent results on care processes and patient outcomes when evaluated in randomized controlled trials. For example, a CDSS generating guideline-based reminders for appropriate antihypertensive drug classes improved medication selection but did not affect patients’ blood pressure [6]. Asthma care improved when providing guideline-based decision support, patient-specific alerts, and a referral option if asthma lacked control [7]. In contrast, an electronic health record (EHR)-based CDSS for diabetes which suggested individually tailored therapeutic changes, laboratory tests, and adequate follow-up intervals optimized glucose and systolic blood pressure control only marginally [8].

Besides the conceptual problem that the current CDSSs typically focus on a single chronic disease and do not address complexity, studies identified various technological, human, and organizational barriers for their acceptance. Technological barriers comprise poor system usability and user-friendliness, while human barriers relate to the lack of computer skills and physicians’ concerns regarding the loss of autonomy in clinical reasoning and/or decision-making power. Also, they are concerned about negative effects on the physician-patient relationship [9–11]. On the organizational level, physicians worry that systems lack integration into workflows but create additional workload [10, 11]. Recently, these drawbacks shifted the approach in health information technology development towards the early involvement of the target physician population [9, 11].

This manuscript presents the study design for the development and feasibility testing of the health care management software eCare*Seniors© which will be designed as a CDSS for the long-term, comprehensive, evidence-based, and individualized health care management of multimorbid seniors. Integrating various strategies and functionalities, this CDSS will assist primary care physicians and practice teams in care processes. The idea and the initial concept were developed and tested as a practice-specific solution in one model practice [12].

Extending the prior concept, the purpose of this study is the development and feasibility testing of a new primary care-defined, EHR-based, software for the long-term management of seniors. The objectives are to (a) further develop the CDSS based on criteria according to the needs of primary care physicians and practice assistants, (b) test the feasibility of the new CDSS in real-life primary care by predefined outcome measures in a mixed-methods study, (c) offer a process evaluation addressing barriers and facilitators for implementation of the CDSS, and (d) provide a description of the CDSS to be tested in a future confirmatory study.

## Methods/design

### Study design

The study is designed as a mixed-methods study in primary care academic teaching practices of the University of Duisburg-Essen, Germany. Following recommendations on health information technology usability evaluation and the framework for the development of complex interventions, the software will be developed in three steps [13, 14]:

- Step 1: specification of the needs for the setting and users
  A series of focus group sessions with primary care physicians and practice assistants as well as practice observations will be conducted to determine setting- and user-specific requirements. (Note: in the German health care system, the term ‘practice assistant’ refers to practice personnel who are typically graduates from a certified 3-year vocational training. They assume tasks in practice organization and patient management).
- Step 2: software development
  Based on the concept developed in the model practice and the requirements identified in the focus groups, a software prototype will be designed. This will be pretested by primary care physicians and practice assistants with subsequent modifications as indicated by the pretesting.
- Step 3: integration of the software into the setting
  To test the new software prototype in routine primary care, a feasibility study will be performed in real-life practice scenarios. If the feasibility testing indicates the need for modification, the software will be refined accordingly.

**Fig. 1:**
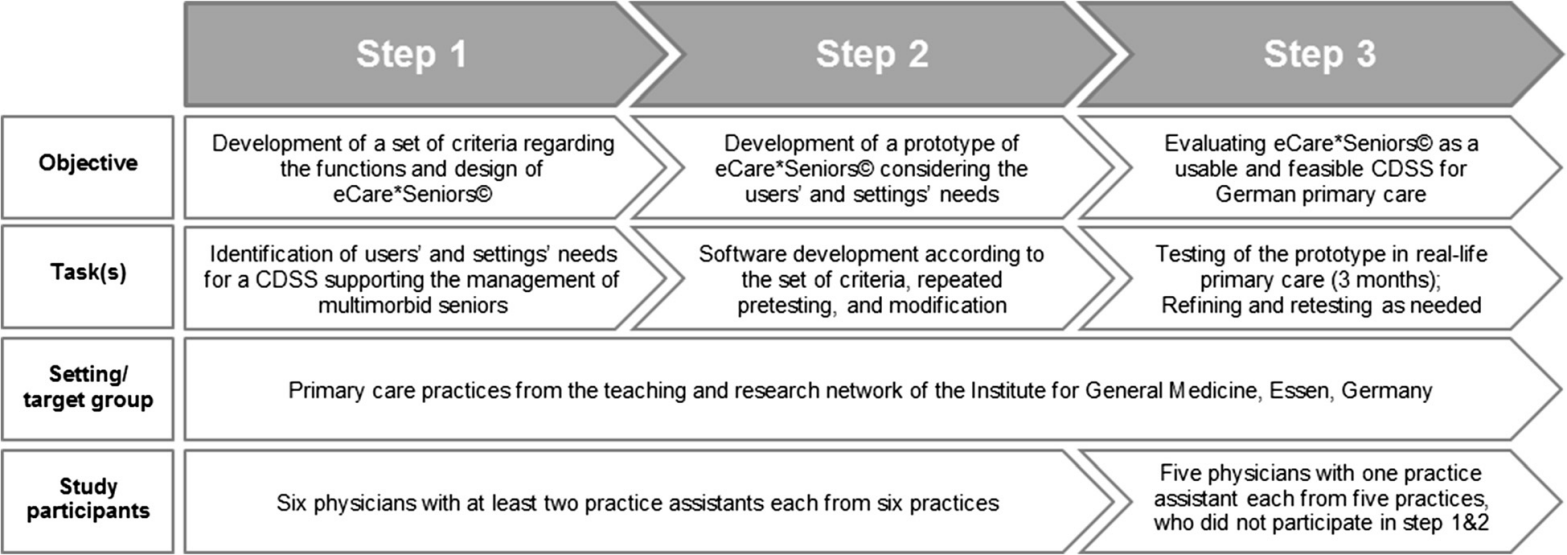
Flow chart of the study conduct

Figure 1 provides an overview of the objectives, tasks, and target groups for the three steps.

### Concept of the health care management software

The software eCare*Seniors© aims at overcoming deficits of the current software solutions for primary care. The logic follows the typical process of health care management for multimorbid seniors in primary care: (1) compilation and updating of all health care and health care process-related aspects in three categories: chronic care, prevention, communication, and organization [12]; (2) evidence-based assignment of priorities based on patients’ quality of life and mortality aspects; (3) integration of patients’ preferences by the assignment of health care priorities in agreement with the patient; (4) long-term individualized health care planning; and (5) support of everyday decision-making and practice processes for individualized health care management. The software solution aims at supporting German primary care physicians typically working in physician-owned practices with one to five physicians yet is applicable to other settings as well. The software is designed as a patient-centered, workflow-oriented platform which integrates responsibilities of physicians and practice assistants. The concept of the software solution integrates components of the chronic care model [15–17] and aims at practice redesign with optimized care processes for multimorbid seniors.

### Description of the user interface

The software has two key features: a physician module (physician control center) and a patient-centered visualization strategy (patient management center).

The first feature is a control center for the physician(s) responsible for practice management and the design of care processes. This control center is important to address potential physicians’ concerns regarding a loss of autonomy in clinical reasoning and/or decision-making power. The software offers choices for various settings in a higher-level structure which are based on epidemiologic data from our prior study, literature data on seniors’ care needs, and evidence-based recommendations. The options refer to the patients to be managed with the software (e.g., if the software is used for all seniors or special subgroups only), the spectrum of outcome-relevant conditions selected for management (e.g., if a physician prioritizes hypertension and diabetes care, while excluding other diseases), the degree of comprehensiveness (e.g., if disease-related and other aspects of care such as the availability of advanced directives or the involvement of a nursing service are included), the level of detail used for the patient-centered visualization (e.g., if chronic renal failure is presented as general information or detailed according to the stage of renal failure). An electronic tutorial will inform physicians about the software and its options and guide through the selection process for practice-specific configurations. This physician control center can be accessed at any time to newly define choices either by adding, refining, or removing options. For practices starting to use the software, an outcome-oriented ‘standard configuration’ is suggested. As the software leads to standardization and redesign of practice processes, it is important that the physician(s) in charge can determine the content and time sequence of quality processes suggested by the software. This helps to avoid excessive workloads and subsequent frustration of physicians and personnel using the software. For better system control in larger health care settings, a directory of setting-specific users’ rights and access codes will be integrated into the physician control center. The rights to access and edit information within the software will thus be structured hierarchically: physicians as administrators will define custom settings in the physician control center and assign user-specific rights depending on the professional and their individual role in practice care processes.

**Fig. 2:**
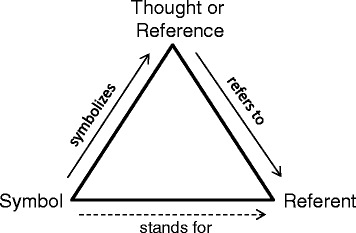
Model of the semiotic triangle

**Fig. 3:**
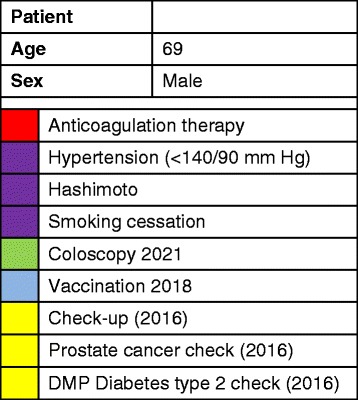
Example of the patient-centered visualization strategy of eCare*Seniors© with information flags (chronic diseases, medication) and dynamic action flags (upcoming preventive and therapeutic measures)

The second key feature of the software is the patient management center which applies a patient-centered visualization strategy: the various aspects of patients’ care will be highlighted in a new graphical user interface with symbols which are a combination of a colored field and a short keyword (so-called flags). This strategy is based on the semiotic triangle (Fig. 2) [18], a concept which is well-known in the field of linguistics. Two types of flags will be used. Information flags will provide relevant, patient-centered information, e.g., relevant diagnoses and medications. Dynamic action flags will indicate quality deficits (e.g., if patients’ blood pressure is not controlled) and refer to upcoming preventive and/or therapeutic measures (e.g., preventive measures, monitoring of a medication blood level). Figure 3 provides an example for the patient-centered visualization strategy.

The information source for the flags is each patient’s EHR: copied information will be exported and then newly edited using this flag system. The software will not evaluate or interpret any patient data by itself. All flags, including priorities and triggers for dynamic flags, will be selected by physicians themselves, either on an individual (one patient) or group (multiple patients) level. Depending on the rights assigned by the administrator, practice assistants will be entitled to apply dynamic flags for individual care processes in the patient management center, e.g., check-up or immunization intervals. When opening a patient’s EHR, the user interface of the software will automatically pop up in a separate window and provide the physician-selected flags for this individual patient’s health care management.

### Technical classification as CDSS

Depending on the human-computer interaction, a CDSS can undertake different functions in the process of decision-making: reminder, advisor, critic, or pilot [11, 19, 20]. Our software will integrate the four CDSS functions:

- It will visualize relevant aspects of each patient’s care using the information flags (reminder).
- It will provide physician-specified dynamic action flags for age- and sex-specific preventive measures, for diagnosis-specific measures (e.g., follow-up care after cancer treatment), and/or for predefined patient groups (advisor).
- It will point at quality deficits by providing dynamic action flags, e.g., if patients’ blood pressure is not controlled (critic).
- It will intuitively guide the user through the system (pilot).

Similar to other CDSSs, eCare*Seniors© combines the characteristics of an individually tailored approach and the characteristics of a population-based quality management system [11].

### Conduct of the study

#### Step 1: specification of the needs for the setting and users

##### Practice recruitment

The first step of the study will be conducted in the teaching and research practice network of our institute consisting of 185 primary care practices located in North Rhine-Westphalia, Germany. All practices are requested to take part in one of the two network meetings of the institute yearly. At one of these practice meetings, physicians will be informed about the study which requires the participation in two focus group sessions and one practice observation. Physicians who are interested in participating will be asked to sign up during the meeting. Interested physicians will be contacted by phone. For the focus groups and practice observations, we will recruit six physicians with up to two practice assistants each. This number is reasonable because the practices are similar to other practices in size, aim, and content of their work [21] belonging to the Association of Statutory Health Insurance Physicians North Rhine and Westphalia-Lippe. No inclusion and exclusion criteria for the practices will be defined.

##### Definition of CDSS criteria

First, a systematic literature search on recent studies including systematic literature reviews and meta-analyses will be conducted to identify CDSS functions described as effective in prior studies and to identify barriers and facilitators for using CDSSs in primary care practices. This information is needed to circumvent ineffective functionalities and to adequately address potential barriers for software utilization. Additionally, a search on population-based disease statistics and on guidelines for the treatment of common chronic diseases will be conducted. This is needed to program the various information and action flags within the software solution.

Second, two focus group sessions with primary care physicians and practice assistants from our practice network and practice observations will be conducted. The focus group sessions will be carried out in a mixed disciplinary group, i.e., physicians and practice assistants together, with a maximum of 15 persons per group. They will be facilitated by physicians and research team members of our institute. The current concept of eCare*Seniors© will be presented and discussed to identify requirements necessary to provide long-term optimized care for multimorbid seniors. Both focus group sessions will be audio-recorded.

Third, additional structured practice observations of everyday practice routines will be performed in the practices of those physicians participating in the focus group sessions. The observations will be carried out by a research team member of our institute and aim at better understanding which patient-related processes need to be addressed in the software. Based on a fictitious clinical vignette of a multimorbid senior, it will be documented whether the practices use specific markers in the EHR to track defined contents of care and patient characteristics, what kind of markers they use, how they are reminded of special contents of care, and who is responsible for applying and updating such markers. These practice observations will be recorded on a semi-structured documentation sheet which combines a simple checklist with free text items allowing for detailed comments on practice specifics.

##### Data analysis

Audio-recorded data of the focus groups will be transcribed. Structuring and coding of the focus group data and the free text items from the process observations will be performed in NVivo qualitative data analysis software, Version 10 (QSR International Pty Ltd., 2012). The coding scheme will be developed and refined over time by identifying categories directly from the transcripts and free text items. Both will be analyzed by two researchers. Quantitative data from the practice observations will be analyzed using simple frequency calculations. All statistical analyses will be performed using IBM SPSS Statistics for Windows, Version 22.0 (Armonk, New York: IBM Corp.).

The results from the focus groups and the practice observations will be specified in a set of criteria detailing the primary care requirements regarding the physician module and the patient-centered visualization strategy of the software. These criteria will provide the basis for the development of a prototype of eCare*Seniors©.

#### Step 2: software development

##### Software development

Based on the set of criteria defined in step 1, a prototype of the software will be developed by a company specialized in developing health information technology. The software will include an electronic interface which allows for interoperability with all certified practice administration software solutions available in Germany.

##### Pretesting

The software prototype will be tested by research team members of our institute and by the physicians and practice assistants who participated in step 1 of the study. The pretest will take place at our institute in Essen, Germany. Each participant will test the prototype for ten fictitious multimorbid seniors.

##### Data collection

After pretesting, each participant will complete the validated system usability scale (SUS) [22] providing information on software usability, practicability, and user-friendliness. Additionally, information regarding software aspects that need to be improved will be provided using free text items.

##### Data analysis

The usability of the prototype will be analyzed by determining the SUS score. The score can assume values between 0 and 40. To interpret the result in percent, each score will be multiplied by the factor 2.5 [22, 23]. Single item scores and the average overall score will be calculated. According to the literature, an overall SUS score ≥70 % denotes good usability, while a score ≤50 % indicates a considerable need for improvement [23]. Pretesting will be judged as successful when a SUS score of ≥70 % is reached. The free text items will be structured in NVivo qualitative data analysis software, Version 10 (QSR International Pty Ltd., 2012). The items will be analyzed using a coding scheme which will be developed and refined over time by identifying categories directly from the free text answers. Simple frequencies will be calculated for these categories. All statistical analyses will be performed using IBM SPSS Statistics for Windows, Version 22.0 (Armonk, New York: IBM Corp.).

##### Software modification

If the SUS score and/or the free text items indicate the need to improve the prototype, a set of criteria for software revision will be defined based on these results. The prototype will be modified accordingly. Afterwards, researchers from our institute will check whether all modifications listed in the set of criteria have been adequately implemented and will finally approve the prototype. If not, the procedure of adapting the software and checking the modifications will be repeated until all criteria defined have been adequately addressed.

A written user manual illustrating the usage and functions of eCare*Seniors© will be created.

#### Step 3: integration of the software into the setting

##### Practice and patient recruitment

The feasibility study will be performed to test the newly developed prototype of eCare*Seniors© in real-life primary care. Five practices of our teaching and research practice network (two single practices and three group practices) which did not participate in study steps 1 and/or 2 will be included in this third step of the study. These practices will be randomly chosen from all primary care academic teaching practices of our network. The practices will be asked for study participation by phone. The practice owner and one responsible practice assistant will participate in the study. The software prototype will be installed during an on-site visit of a research team member. The physicians and the practice assistants will be trained on how to use the software and will receive the written user manual. Subsequently, they will consecutively set up eCare*Seniors© for one scheduled patient per day, until it is implemented for 30 patients of the practice. This number is based on the recommendation for sample sizes in pilot studies by Browne [24]. Because the feasibility study is not conducted to estimate effect sizes on practice and/or patient outcomes but to test for the software’s usability in routine primary care, no sample size was calculated.

##### Inclusion and exclusion criteria for patients

The practices will be asked to use eCare*Seniors© for 30 patients aged ≥65 years with more than one chronic disease (multimorbidity) who are taking at least five different chronic medications (polypharmacy).

According to the European General Practice Research Network (EGPRN), multimorbidity is defined as any combination of chronic disease with at least one other acute or chronic disease, or associated or non-associated bio-psychosocial factor, or somatic risk factor (e.g., smoking behavior, chronic job stress, diabetes, heart failure) [2]. We adapted this definition because eCare*Seniors© will be designed to support chronic care management: all seniors with two or more chronic diseases will be eligible for the study.

##### Outcome measures and data collection

The primary outcome of the feasibility study is the usability of eCare*Seniors© which will be measured with the SUS [22] after 3 months of using eCare*Seniors©. The secondary outcomes at 3-month follow-up are the following:

- Willingness to routinely use the software,
- Degree of utilization of eCare*Seniors©,
- Acceptance of eCare*Seniors©,
- Willingness of primary care physicians to purchase eCare*Seniors©
- Willingness of practice assistants to use eCare*Seniors© in the long term.

Data will be collected at baseline and after 3 months. At baseline, the data collection will be conducted by a research team member of our institute during the first on-site visit, before installing and explaining the software. All participating physicians and practice assistants will be surveyed using two approaches:

1. Semi-structured interview (in person) about the practice-specific health care management of multimorbid seniors and how their current practice administration system is supporting these processes. The interviews will be audio-recorded and provide information about practice-related specifics of managing multimorbid seniors.
2. Written questionnaire about socio-demographic characteristics and the participant’s computer literacy (validated computer literacy scale (CLS) [25]). These data will be used to characterize the study participants.

After 3 months, a second on-site visit will be conducted for follow-up data collection consisting of three elements. Again, all participating physicians and practice assistants will be surveyed:

1. Semi-structured interview (in person) about the utilization of eCare*Seniors© in the practice. The interviews will be audio-recorded and provide details about the software’s acceptance, the satisfaction with the software, perceived barriers, ideas for improvement, its planned further utilization (practice assistants’ perspective), and the willingness to purchase the software (physicians’ perspective).
2. Validated SUS [22] about the experienced usability of the new software for the primary outcome measurement.
3. Retrieval of the usage data of eCare*Seniors© in each practice, which will be used to determine the frequency and continuity of using the software during the previous 3 months.

##### Data analysis

The primary outcome of the feasibility study, defined as usability of eCare*Seniors©, will be analyzed by determining the SUS score as described in the outline of step 2. The feasibility study will be judged as successful if a SUS score ≥70 % is reached, denoting good usability [23].

The secondary outcome parameters willingness to purchase the software, willingness for continued use of the software, and utilization usage of the software will be determined by calculating the frequencies. These data will be used to offer insights into the attitudes of users regarding the utilization of eCare*Seniors© for everyday patient management.

Audio-recorded data of the interviews will be transcribed. Structuring and coding of the qualitative interview data will be performed in NVivo qualitative data analysis software, Version 10 (QSR International Pty Ltd., 2012). The coding scheme will be developed and refined over time by identifying categories directly from the transcripts. The transcripts will be analyzed by two researchers. Experiences, attitudes, and/or attributes described in the first and in the second interview will be compared to offer insights into reported advantages and disadvantages of eCare*Seniors© compared to the current practice-specific health care management.

All statistical analyses will be performed using IBM SPSS Statistics for Windows, Version 22.0 (Armonk, New York: IBM Corp.).

##### Software refinement

If the SUS score and/or the content analyses of the interviews indicate the need to improve eCare*Seniors©, these results will be summarized in a set of criteria which will provide the basis for revision. The revised software prototype will be tested in the practices that participated in the feasibility study. Each participant will test the refined prototype for ten patients of the practice as well as complete the SUS and additional free text items regarding improvements and deteriorations compared to the previous version of the prototype. The SUS and the free text items will be analyzed as described previously to check whether the revised prototype fulfills all aspects according to the predefined set of criteria. If not, this procedure will be repeated until all criteria are met.

### Quality controls and data management

Quality controls will be conducted during all steps of the study. After recruitment, consent forms of all study participants will be checked for completeness. After each data collection, quality controls will be conducted to ensure that required data collection documents are complete. An identification number and the date of birth will be used to verify that study data of each study participant are merged correctly.

Audio-recorded data of the interviews and the focus group sessions will be transcribed. Transcripts will be imported into NVivo qualitative data analysis software, Version 10 (QSR International Pty Ltd., 2012). Quantitative data will be entered manually in an access-restricted electronic database. To control for input errors, 10 % of data will be entered twice. An error rate of 5 % will be accepted; otherwise, all values will be entered twice. The data will be checked for plausibility using simple frequency testing.

All data will be stored access-restricted at our institute.

### Ethical considerations

The study conduct complies with the ethical principles of the World Medical Association Declaration of Helsinki [26]. Ethical approval was obtained from the Ethics Commission of the Medical Faculty of the University of Duisburg-Essen (reference number: 14-5980-BO, date of approval: 01/06/2015). All participating primary care physicians and practices will sign an informed consent form which will be stored at our institute. Throughout the feasibility study, physicians will be free regarding the extent to which they implement eCare*Seniors© in patient care. The responsibility for the patients’ care and health care planning remains solely in the hands of the physician in charge.

## Discussion

This study aims at designing a user-friendly, easy-to-comprehend, workflow-oriented, and comprehensive EHR-based CDSS for primary care physicians and non-medical professions which provides assistance in managing multimorbid seniors. Assuring evidence-based and individual health care for seniors is a key future challenge for physicians given the decreasing resources in the health care sector, the aging population with increasing multimorbidity, evolving diagnostic and therapeutic options, the trend towards an information society with patients who are involved in medical decision-making, and the subsequent diversity of patients’ preferences. Consequently, a well-designed electronic decision support system—such as the one we aim for with eCare*Seniors©—will be relevant to ensure seniors’ comprehensive long-term care.

eCare*Seniors© addresses a health care management problem in a target group of increasing importance in many societies. Potential barriers of CDSS acceptance identified in prior studies [9–11] will be addressed using a bottom-up approach focusing on the end user and the target setting:

1. Aiming at a user-friendly and easy-to-comprehend software solution, end users will be continuously involved in all steps of the software design: the needs of primary care physicians and practice assistants to manage multimorbid seniors will be assessed using focus groups and process observations, the software will be tested in real-life primary care, and repetitive practice tests will be conducted until the software is fully adapted to primary care needs.
2. The continuous involvement of the end users’ perspectives will also contribute to optimal workflow integration of eCare*Seniors©: organizational needs can be identified promptly and taken into account for software design. To prevent additional workload, eCare*Seniors© will allow for setting-specific tailoring of the software: physicians will select which components and functions they want to use for their patient population as well as individual patients. The physician control center as an integral element of the software will support physician managers in improving their settings’ health care for seniors over time.
3. With the objective of maintaining the physician’s autonomy, eCare*Seniors© will provide passive decision support only: the software will offer options but will not replace the physician’s decision-making. The physician control center helps the user to preselect practice-relevant components with relevant information and action flags. Based on this preselection, the software will only suggest options if desired by the physician.
4. To address concerns about negative effects on the physician-patient relationship, eCare*Seniors© will allow for a patient-centered and physician-selected use of the software according to physician and patient priorities: all patient-related flags will be selected by physicians so that patients’ preferences as well as physicians’ priorities can be integrated in the care for each individual patient.
5. For optimal integration into organizational structures and workflows, eCare*Seniors© is designed as a platform with access for physicians and non-medical professionals. According to studies which show that practice assistants are a valuable resource in reducing primary care physicians’ workload, increasing efficiency, and improving chronic care patient management [27, 28], eCare*Seniors© will be designed as a comprehensive, interdisciplinary communication platform allowing for the short- and long-term management of individual patients as well as larger patient groups. This includes the integration of recall options for relevant care processes. In each practice, one physician will serve as an administrator and manage access and editing rights depending on each person’s profession and role within the practice.
6. A comprehensive evaluation of the software based on the latest frameworks available for the evaluation of health information technology will be conducted [14, 29].

After the final approval of eCare*Seniors©, we will aim to demonstrate the software’s effectiveness on seniors’ outcomes in a cluster randomized trial with a larger group of primary care practices.

## Abbreviations

CDSS: clinical decision support system
CLS: computer literacy scale
EHR: electronic health record
SUS: system usability scale
